# Primary motor cortex and fast feedback responses to mechanical perturbations: a primer on what we know now and some suggestions on what we should find out next

**DOI:** 10.3389/fnint.2014.00072

**Published:** 2014-09-15

**Authors:** J. Andrew Pruszynski

**Affiliations:** Department of Integrative Medical Biology, Physiology Section, Umeå UniversityUmeå, Sweden

**Keywords:** reflex, long-latency, upper-limb, primary motor cortex, transcortical pathway

## Abstract

Many researchers have drawn a clear distinction between fast feedback responses to mechanical perturbations (e.g., stretch responses) and voluntary control processes. But this simple distinction is difficult to reconcile with growing evidence that long-latency stretch responses share most of the defining capabilities of voluntary control. My general view—and I believe a growing consensus—is that the functional similarities between long-latency stretch responses and voluntary control processes can be readily understood based on their shared neural circuitry, especially a transcortical pathway through primary motor cortex. Here I provide a very brief and selective account of the human and monkey studies linking a transcortical pathway through primary motor cortex to the generation and functional sophistication of the long-latency stretch response. I then lay out some of the notable issues that are ready to be answered.

## Introduction

The nervous system responds to unexpected mechanical perturbations with a stereotypical sequence of muscle activity. The fastest and crudest response is the short-latency stretch response, which occurs so quickly that is must reflect spinal processing (Pierrot-Deseilligny and Burke, 2005). The slowest and most sophisticated response is labeled “voluntary”, often because it occurs at latencies greater than typical measures of voluntary reaction time (Prochazka et al., 2000). At intermediate latencies is the long-latency stretch response, which occurs faster than typical measures of voluntary reaction time yet produces a wide range of sophisticated responses often reserved for voluntary control processes (for reviews, see Scott, 2004, 2012; Shemmell et al., 2010; Pruszynski and Scott, 2012): modulation by subject intent (Hammond, 1956; Hagbarth, 1967; Crago et al., 1976; Evarts and Granit, 1976; Colebatch et al., 1979; Rothwell et al., 1980; Pruszynski et al., 2008; Shemmell et al., 2009; Manning et al., 2012; Ravichandran et al., 2013), sensitivity to task goals (Marsden et al., 1981; Doemges and Rack, 1992a,b; Dietz et al., 1994; Häger-Ross et al., 1996; Nashed et al., 2012), engagement during decisional processes (Yang et al., 2011; Selen et al., 2012; Nashed et al., 2014), flexible routing of sensory information across the musculature (Cole et al., 1984; Ohki and Johansson, 1999; Mutha and Sainburg, 2009; Dimitriou et al., 2011; Omrani et al., 2013), and knowledge of the physical properties of the arm (Gielen et al., 1988; Soechting and Lacquaniti, 1988; Koshland et al., 1991; Kurtzer et al., 2008, 2009, 2013, 2014; Crevecoeur et al., 2012; Crevecoeur and Scott, 2013) and environment (Akazawa et al., 1983; Bedingham and Tatton, 1984; Dietz et al., 1994; Kimura et al., 2006; Perreault et al., 2008; Pruszynski et al., 2009; Shemmell et al., 2009; Krutky et al., 2010; Ahmadi-Pajouh et al., 2012; Cluff and Scott, 2013).

Here, I provide a brief review of the monkey and human studies linking the long-latency response of the arm, and its functional sophistication, to a transcortical pathway centered on primary motor cortex (M1). Understanding these neural links is motivated by recent theories of motor control—based on optimal feedback control (Todorov and Jordan, 2002)—which suggest that voluntary motor behavior reflects sophisticated manipulation sensory feedback (Scott, 2004). My intention is not to be exhaustive (for that, see Pruszynski and Scott, 2012), but rather to highlight a few particularly notable studies to summarize what we know now and motivate a few things that we should do next.

## Transcortical contribution to the long-latency stretch response

**Figure 1:**
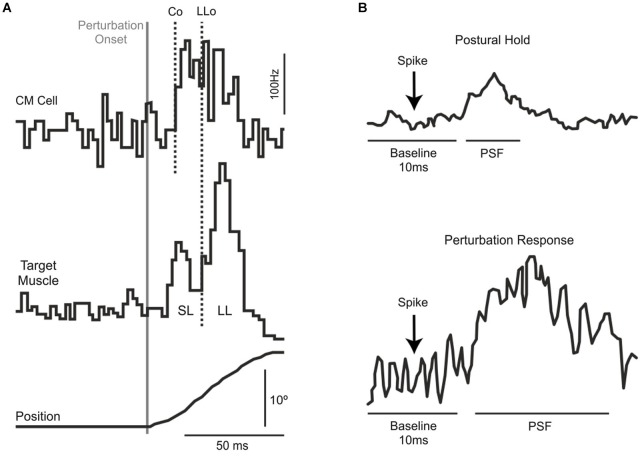
**(A)** Extracellular recordings from a single corticomotoneuronal neuron in primary motor cortex (top) and muscle activity from its target muscle (middle) in response to excitatory torque perturbations that causes wrist displacement (bottom). Traces are aligned on perturbation onset. The vertical lines represent the onset of the CM activity (Co) and the onset of the long-latency response in the target muscle (LLo). **(B)** Spike-triggered averages in the target muscle during postural maintenance (top) and after mechanical perturbation onset (bottom). Note the increased post-spike facilitation during the perturbation epoch. Panels **(A)** and **(B)** modified with permission from Cheney and Fetz (1984).

There are essentially three independent lines of evidence—in monkeys and humans–that a transcortical pathway though M1 contributes to the long-latency stretch response. The first and strongest evidence comes from monkey work showing that corticomotoneurons, which project directly from M1 to motoneurons, produce post-spike facilitation in their target muscles at such short latencies (Figure 1A) that they can contribute to the long-latency stretch response even when accounting for sensory delays (Cheney and Fetz, 1984). Moreover, the observed post-spike facilitation is stronger for spikes occurring during a mechanical perturbation than for spikes occurring during a static hold period (Figure 1B), indicating that the causal effect of action potentials from corticomotoneurons in M1 is particularly potent during the long-latency response to mechanical perturbations. These findings are supported by a range of studies in both humans (Abbruzzese et al., 1985; MacKinnon et al., 2000; Spieser et al., 2010) and monkeys (Evarts and Tanji, 1976; Fromm and Evarts, 1977; Wolpaw, 1980; Picard and Smith, 1992; Pruszynski et al., 2011a, 2014) showing changes in M1 activity following perturbation onset that precede the long-latency stretch response.

The second line of evidence comes from clinical studies of people who suffer from Kippel-Fiel syndrome, which causes undesired bilateral movements because of a bilateral bifurcation of descending projections from M1 to the spinal cord. When these participants are presented with mechanical perturbations applied to the finger, they demonstrate unilateral short-latency stretch responses but bilateral long-latency responses (Matthews et al., 1990; Capaday et al., 1991). Specifically, a mechanical perturbation that stretches finger muscles on one hand yields short-latency stretch responses only in the stretched finger muscles but yields long-latency stretch responses in both the stretched finger muscles on that hand and the same (unstretched) finger muscles on the other hand. Because the motor pathway in this patient group bifurcates at the level of M1 output, the mapping from stretched muscle inputs to unstretched muscle outputs must have occurred at that level of M1 or above.

The third line of evidence comes from brain stimulation studies. A wide range of work has shown a supra-linear interaction between the long-latency stretch response elicited by a mechanical perturbation and transcranial magnetic stimulation applied over M1 (Day et al., 1991; Palmer and Ashby, 1992). The most likely explanation for this interaction, which does not occur for the short-latency response, is that the neural mechanisms generating the long-latency stretch response and magnetic stimulation are physically co-localized at the site of stimulation, that is, M1.

## Functional modulation in primary motor cortex

Several studies have observed flexible responses in M1 neurons to mechanical perturbations applied to the limb (Evarts and Tanji, 1976; Fromm and Evarts, 1977; Wolpaw, 1980; Picard and Smith, 1992; Pruszynski et al., 2011a, 2014). The most notable of these is the seminal work of Evarts and Tanji (1976) who trained monkeys to respond to a mechanical perturbation by either pulling or pushing the perturbing handle to its limits. They found that M1 neurons signaled the instructed action (Tanji and Evarts, 1976) and then subsequently responded to the same perturbation with two distinct components (Evarts and Tanji, 1976). First, there was a relatively short-latency response starting ~20 ms after perturbation onset that showed little or no modulation according to the instructed action and a second component starting ~40 ms post-perturbation which was sensitive to the prior instruction. This timing appeared early enough to account for a clear goal-dependent response in arm muscles starting about 70 ms following perturbation onset.

We recently extended this study to show that such modulation holds when the monkey is performing a task that more closely mirrors previous human work (Pruszynski et al., 2014). Most notably, our task used spatial goals that yielded behavioral responses analogous to the typical “resist” and “do not intervene” verbal instructions and we ensured that the muscles stretched by the mechanical perturbation were pre-activated by a tonic load (Pruszynski et al., 2008). The latter control is particularly critical as it ensures that any change in muscles activity—known to modulate the long-latency stretch response—would be above threshold and thus could be observed (Bedingham and Tatton, 1984; Matthews, 1986; Pruszynski et al., 2009). Our findings were largely consistent with Evarts and Tanji (1976). We found that monkey muscles, like human muscles, showed a multi-phasic response with goal-dependent starting about 70 ms after perturbation onset. And we also noted that the initial response of M1 neurons—which began around 20 ms post-perturbation—was not sensitive target position and that goal-dependent activity in M1 neurons emerged about 40 ms after perturbation onset. However, our paradigm revealed a great deal of additional complexity, including the striking observation that many neurons changed their preference from one goal target to another over time following the perturbation.

**Figure 2:**
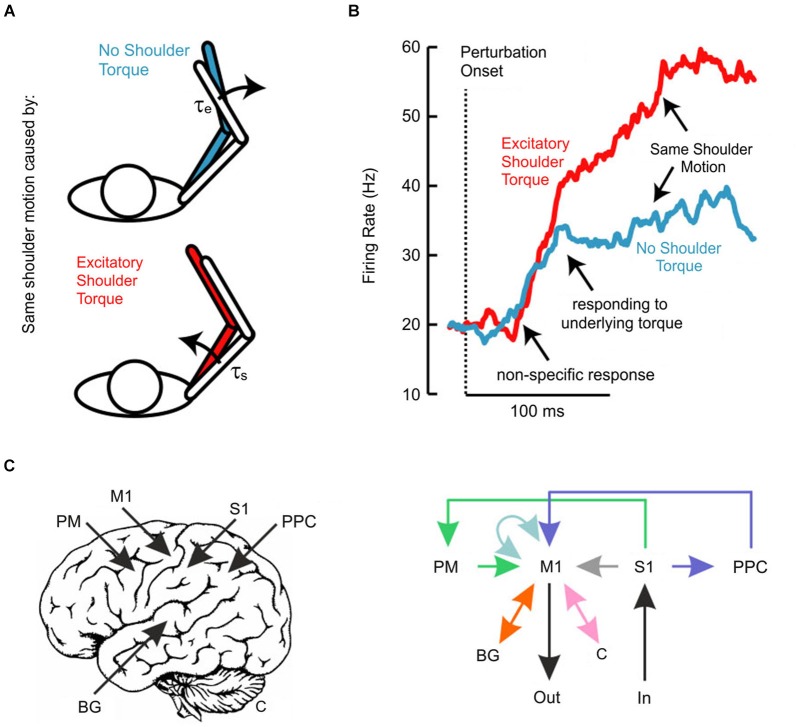
**(A)** Schematic of experiment investigating whether long-latency responses account for limb dynamics. Perturbations were chosen so that the same shoulder motion arose because of either a pure shoulder or pure elbow torque. **(B)** Traces depict the average population response of neurons in primary motor cortex aligned on perturbation onset. Note that the two conditions evoke the same initial response and that appropriate differentiation does not emerge until ~50 ms post-perturbation. **(C)** Schematic representation of the neural pathways that likely contribute to the long-latency response in primary motor cortex. Deciphering which of these circuits contributes under which circumstances is an important outstanding question. Panels **(A)** and **(B)** modified with permission from Pruszynski et al. (2011a).

We have also recently investigated whether the transcortical feedback pathway allows the long-latency stretch response to account for the mechanical properties of the limb (Pruszynski et al., 2011a). In this study, we applied mechanical perturbations at the shoulder and/or elbow joints (Kurtzer et al., 2008, 2009, 2014) to examine whether and when neurons in monkey M1 responded to the underlying torque as opposed to the resulting motion, factors which are decoupled because of the intersegmental dynamics of the limb (Figure 2A). Strikingly, the earliest response did not distinguish between the various loading conditions and such discrimination began 40–50 ms after perturbation onset (Figure 2B), still about 20 ms before arm muscles appropriately responded to the applied shoulder load. Since local joint motion itself provides ambiguous information about the underlying shoulder torque and since the only other piece of available information arises at the other joint, these findings indicate that M1 neurons eventually integrate elbow and shoulder motion to identify and counter the applied torque, which must be done to stabilize the limb. Notably, we also established a causal role for M1 by applying TMS over human M1 and showing that the long-latency stretch response in shoulder muscles was potentiated even when the shoulder joint was not displaced by the mechanical perturbation. As described above, such potentiation (Day et al., 1991; Palmer and Ashby, 1992; Lewis et al., 2004) must reflect the impact of elbow afferent information onto a cortical circuit controlling shoulder muscles since local shoulder afferents are not physically affected by the perturbation.

Indeed, several studies have used TMS to link M1 to specific functional capabilities of the long-latency response (Kimura et al., 2006; Shemmell et al., 2009; Spieser et al., 2010). In a very elegant study, Kimura et al. (2006) showed that disrupting sensorimotor cortex did not completely abolish the long-latency response; rather, the stimulation specifically impaired the ability of the long-latency response to predictively compensate for external force fields during reaching. The same approach has been used by Shemmell et al. (2009) to show that interfering with M1 does not change long-latency activity associated with the verbal instructions given to the subject but does affect long-latency activity associated with the stability of the environment, suggesting that only the latter functionality relies on a circuit that includes M1.

## What we should find out soon

As a basis for motivating future work, it is worth quickly reemphasizing what we know today. We know there exists a phasic epoch of muscle activity—termed the long-latency stretch response—that occurs prior to standard measure of voluntary reaction time (Hammond, 1955; Pruszynski et al., 2008). We know that M1 contributes to the long-latency response under normal circumstances (Cheney and Fetz, 1984; Matthews et al., 1990; Capaday et al., 1991; Day et al., 1991; Palmer and Ashby, 1992) but that M1 is not required for observing activity in the long-latency epoch (Tracey et al., 1980; Miller and Brooks, 1981). We know that the long-latency stretch response exhibits a host of sophisticated capabilities during both posture and movement (for detailed review, see Pruszynski and Scott, 2012). And we know that some of these sophisticated responses are apparent in M1 (Evarts and Tanji, 1976; Fromm and Evarts, 1977; Wolpaw, 1980; Picard and Smith, 1992; Kimura et al., 2006; Shemmell et al., 2009; Spieser et al., 2010; Pruszynski et al., 2011a, 2014).

We don’t know the limits of the sophistication of the long-latency stretch response relative to voluntary control. Given recent work, however, it is tempting to speculate that the long-latency response exhibits all the capabilities of voluntary motor control within the constraints imposed by processing time. For example, recent work shows that the long-latency stretch response is modified as subjects learn novel force environments (Ahmadi-Pajouh et al., 2012; Cluff and Scott, 2013) and that those subjects who learn more show more substantial modulation of the long-latency stretch response (Cluff and Scott, 2013). Thus, adapting motor commands to compensate for changes in the environment—often considered a hallmark of voluntary motor control (Shadmehr and Wise, 2005)—at least partly rely on changes in feedback control processes such as the long-latency stretch response. Similarly, we now know that the long-latency stretch response includes predictions about the future state of the limb based on priors about the load environment (Crevecoeur and Scott, 2013). Such a predictive scheme—akin to a forward model (Kawato and Wolpert, 1998)—seems critical for ensuring stable feedback control with noisy and delayed inputs.

One important capacity that has yet to be explored in detail is whether and how the long-latency stretch response accounts for the kinematic redundancy of the limb. That is, if a given motor task can be accomplished in many ways, as it almost always can, does the neural machinery that generates the long-latency response tend to choose solutions that optimize task success? This type of adaptive control has been shown in various contexts for voluntary motor control (Latash et al., 2002; Todorov, 2004) but, so far has only been suggested with respect to the long-latency stretch response (Scott, 2004). We also know little about whether and how the long-latency stretch response integrates multiple pieces of sensory information such as that arising from tactile mechanoreceptors, muscle spindles and Golgi tendon organs. Take, for example, our own result showing that the long-latency stretch response accounts for the dynamics of the limb when generating a shoulder response by integrating motion information across both the elbow and shoulder (Kurtzer et al., 2008; Pruszynski et al., 2011a). The plainest explanation is that this integration is based on sensory information arising from the muscles themselves but it may well be tactile inputs from the stretching skin, which travel as slightly slower transmission speeds, are critical in this respect. Furthermore, the long-latency stretch response is only one of many fast feedback responses that can potentially contribute to muscle activity in the long-latency epoch (Goodale et al., 1986; Pélisson et al., 1986; Pisella et al., 2000; Franklin and Wolpert, 2008; Pruszynski et al., 2010; White and Diedrichsen, 2010; Knill et al., 2011). Understanding the role of these different modalities and, specifically, how they interact and how they are integrated in naturalistic motor behavior (for topical reviews, see Hatsopoulos and Suminski, 2011; Cluff et al., 2014) is critical for our broader understanding of limb motor control.

Functional questions notwithstanding, I believe that most critical outstanding issues relate to how the various neural pathways and circuits help form and sculpt the long-latency stretch response. I have emphasized so far the notion that the sophistication of the long-latency stretch response arises because of a transcortical feedback pathway centered on M1. It is critical to emphasize however, that M1 does not act alone and the transcortical feedback pathway includes potential contributions from many other structures both cortical (e.g., premotor cortex, posterior parietal cortex) and subcortical (cerebellum, basal ganglia) (Scott, 2004). Although less is known about these areas and how they contribute to fast feedback responses as compared to M1, there is plenty to suggest that they do contribute and a key challenge for future studies is to unravel when and how this occurs.

A potential window into this problem may be the repeated observation that the initial phase of M1 activity—starting about 20 ms post-perturbation—appears to be relatively fixed and that sophisticated responses do not arise until about 40 ms post-perturbation (Evarts and Tanji, 1976; Pruszynski et al., 2011a, 2014), even when the required response is known well in advance of the perturbation. Such a non-specific response is similar to neurons in primary visual cortex, which respond quickly to objects in their receptive field but do not signal motion direction for another 20–30 ms, a delay attributed to processing among neurons within primary visual cortex (Knierim and van Essen, 1992). The temporal evolution of the long-latency response may also reflect intrinsic processing in M1 or, perhaps more likely, it may reflect the additional influence of other neural structures (Figure 2C).

One candidate is cerebellum. It is well established that there exist neurons in the dentate and interpositus nuclei of the cerebellum that respond to mechanical perturbations (Strick, 1983) and, indeed, the long-latency stretch response is reduced in humans who suffer cerebellar dysfunction (Hore and Vilis, 1984; Kurtzer et al., 2013). Those neurons in interpositus respond quickly to the perturbation (~20 ms) but have little or no goal-dependent modulation whereas neurons in dentate tend to respond at longer-latencies and are strongly influenced by the goal of the task. It is tempting to suggest that the two distinct components of the long-latency response in M1 reflect inputs from the interpositus and dentate nuclei, respectively. However, this cannot be the full story, as cooling the entire cerebellum leads to little change in the initial response and only partially reduces the second response (Meyer-Lohmann et al., 1975; Vilis et al., 1976). On the other hand, one reasonable hypothesis, as yet untested, is that dentate neurons modulate rather than generate the later M1 response.

There exist other candidate contributors. For example, previous studies have reported that pre-motor cortical neurons quickly respond to mechanical perturbations (Picard and Strick, 1996; Boudreau et al., 2001) and this area, which projects directly to M1, is known to be remarkably sensitive to motor planning and task goals (Picard and Strick, 1996; Wise et al., 1997; Cisek and Kalaska, 2005). Similarly, posterior parietal cortex is involved in attentional mechanisms and motor control (Andersen and Buneo, 2002), receives inputs from somatosensory cortex and projects to the frontal cortex including M1 (Petrides and Pandya, 1984). Diseases of the basal ganglia typically lead to markedly exaggerated long-latency stretch responses (Tatton and Lee, 1975; Rothwell et al., 1983), which may reflect changes in the transcortical pathway (DeLong and Wichmann, 2007), though recent studies with Parkinsonian monkeys suggest that such effects are more complicated than mere changes in the sensitivity of M1 neurons to sensory input (Pasquereau and Turner, 2013). And recently, a compelling argument has been made that startle-like brain stem processes contribute to the long-latency stretch response in various contexts (Shemmell et al., 2010) and, indeed, neurons in the reticular formation that project to the distal arm muscles also respond to mechanical perturbations at such short latencies that they likely contribute to muscle activity in the long-latency epoch (Soteropoulos et al., 2012).

In sum, the long-latency stretch response is strikingly sophisticated and, though most effort has been centered on its generation and modulation via the transcortical pathway through primary motor cortex, it likely involves many neural circuits with their own complex interactions (Kimura et al., 2006; Lourenço et al., 2006; Shemmell et al., 2009; Pruszynski et al., 2011b). Experiments with modern techniques are needed to revolve which of these circuits contribute to which functional capacity under what circumstances, how each pathway accounts for the actions of the others, and how processing for feedback responses relates to the circuitry typically associated with voluntary motor control.

## Conflict of interest statement

The author declares that the research was conducted in the absence of any commercial or financial relationships that could be construed as a potential conflict of interest.
